# Supplementary material for: Association of Prenatal Polycyclic Aromatic Hydrocarbons Exposure, DNA Hydroxymethylation, and Neurodevelopment at 0 and 2 Years of Age
Source: Toxics. 2025 Aug 29;13(9):726. doi: 10.3390/toxics13090726 (PMC12473805; doi:10.3390/toxics13090726)
Supplement: Supplementary file 1 [file toxics-13-00726-s001.zip › toxics-3766688-supplementary.pdf]

Table S1. Primer sequences of BDNF and MeCP2 genes

Gene symbol	Chromosomal location	Primer sequence (5 '-3')
BDNF	chr11:27654893-27720779	F: CCCACCCACTTTCCCATTCA
		R: CGGAGGTAATACTCGCACCC
MeCP2	chrX:154021573-154097755	F: GCCCACTAAACCAGTCCCTC
		R: ACCCCTCCAGCTGTTGATTG

Table S2. Sensitivity analyses

Outcome	1-OHPyr		Biomarker	1-OHPyr	
	OR (95%CI)	P		OR (95%CI)	P
Motor	-4.74 (-8.07, -1.42)	0.005	Global DNA	0.024 (0.01, 0.04)	<0.001
Adaptive	-6.35 (-9.50, -3.20)	<0.001	MeCP2	-6.52 (-9.26, -3.78)	<0.001
Language	-5.93 (-9.72, -2.14)	0.002	BDNF	-3.42 (-5.37, -1.46)	<0.001
Social	-4.81 (-7.94, -1.69)	0.003			

Covariates included mother’s age, mother’s education, prepregnant BMI, household income, parity, passive smoking, child’s sex, gestational age, cord blood lead, 3-OHChr, 6-OHChr, and 9-OHBap.
